# Prediction of acute pancreatitis complications using routine blood parameters during early admission

**DOI:** 10.1002/iid3.747

**Published:** 2022-11-25

**Authors:** Xiubing Chen, Jing Ning, Qing Li, Wenxi Kuang, Haixing Jiang, Shanyu Qin

**Affiliations:** ^1^ Department of Gastroenterology The First Affiliated Hospital of Guangxi Medical University Nanning China

**Keywords:** acute pancreatitis, complications, pancreatic pseudocyst, prediction, routine blood parameters

## Abstract

**Background:**

There have been many reports on biomarkers for predicting the severity of acute pancreatitis (AP), but few studies on biomarkers for predicting complications; some simple and inexpensive indicators, in particular, are worth exploring.

**Methods:**

We retrospectively collected clinical data of 809 AP patients, including medical history and results of routine blood tests, and grouped them according to the occurrence of complications. Differences in clinical characteristics between groups with and without complications were compared using *t*‐test or *χ* ^2^ test. Receiver operating curve (ROC) and area under the curve were calculated to evaluate the ability of predicting the occurrence of complications for the routine blood parameters with statistical differences. Then, through univariate and multivariate analyses, independent risk factors closely associated with complications were identified. Finally, we built a three‐parameter prediction system and evaluated its ability to predict AP complications.

**Results:**

Compared with the group without complications, the patients in the complication group had higher white blood cells, neutrophils, C‐reactive protein, and erythrocyte sedimentation rate (ESR), and lower red blood cells and hemoglobin (Hb) (all *p* < .05), and most of them had severe pancreatitis. In addition, pseudocysts were more common in patients with alcoholic etiology, recurrence, low BMI, and high platelet (PLT) and plateletocrit. Acute respiratory failure was more common in patients with first onset and high mean PLT volume (MPV). Sepsis was more common in patients with lipogenic etiology, high MPV, and low lymphocytes. Infectious pancreatic necrosis was more common in patients with alcoholic etiology. Acute renal failure was more common in patients with monocytes and high MPV and low PLT. Multivariate analysis showed that PLT and ESR were risk factors for pseudocyst development. The ROC showed that the combination of Hb, PLT and ESR had a significantly higher predictive ability for pseudocyst than the single parameter.

**Conclusion:**

Routine blood parameters can be used to predict the complications of AP. A predictive model combining ESR, PLT, and Hb may be an effective tool for identifying pseudocysts in AP patients.

## Graphical abstract

We studied the clinical data and routine blood parameters of 809 patients with acute pancreatitis, and found that there were significant differences in routine blood parameters between patients with and without complications. In addition, we built a three‐parameter model to predict pancreatic pseudocysts.

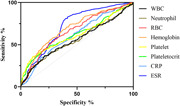

## 1 INTRODUCTION

With improvement in living standards and changes in lifestyle, the incidence of acute pancreatitis (AP) is increasing every year at an annual rate of 3.4%, and the overall mortality rate is about 5%. Among them, acute severe pancreatitis accounts for about 20% of all pancreatitis cases, but its mortality rate is as high as 30%.^1^, ^2^, ^3^, ^4^ The severity of AP varies widely, and the occurrence of complications is often an important cause of prolonged illness, increased hospital stay, and increased mortality.^5^, ^6^ The main complications of AP include pancreatic pseudocyst, acute respiratory failure, sepsis, infectious pancreatic necrosis, acute renal failure.^7^, ^8^, ^9^ In recent years, thanks to early intervention using some new treatment methods, such as fluid resuscitation, endoscopic trans‐luminal drainage, and endoscopic step‐up approach, the progression of pancreatitis and the occurrence of complications have been favorably influenced.^7^ However, available evidence suggests that the benefits of these treatments remain unsatisfactory, that many health care facilities are not equipped to provide these treatments, and that it is financially not feasible for all patients to receive them. Therefore, prediction of the possible complications of AP in advance can help improve the ability of clinicians to manage these patients.

Some predictive models for pancreatitis complications have emerged.^6^, ^10^, ^11^ However, these models either require complex and special laboratory inspection data or cumbersome algorithms, which greatly limit their clinical utility and application. Therefore, there is an urgent unmet need to find an inexpensive, readily available, and sensitive laboratory test to predict complications of AP. A routine blood test is a very simple laboratory test. In the past, it was mainly used for the classification and diagnosis of anemia and the preliminary judgment of the severity of infectious diseases. However, recent studies have shown that routine blood parameters can predict AP severity^12^ and inflammatory bowel disease complications.^13^

In this study, we used routine blood parameters to predict AP complications rather than severity, and combining these with significant independent predictors, we further refined the prediction model for Chinese AP patients. After verification, these simple parameters can replace complex and expensive measurements and become cheap, convenient, and effective predictors of AP complications.

## 2 METHODS

### 2.1 Patients

This retrospective study included 950 patients with AP who were hospitalized in the First Affiliated Hospital of Guangxi Medical University between January 2017 and July 2022. Inclusion criteria: age ≥ 18 years, with a clear diagnosis of pancreatitis after blood biochemical and computed tomography (CT) examinations.^7^ All patients were classified according to the 2012 Atlanta Classification of AP.^14^ Complications of AP were defined and categorized into one of the following groups: pancreatic necrosis, acute peripancreatic fluid collections, acute necrotic collections, acute organ dysfunction, pancreatic pseudocysts.^9^, ^14^, ^15^ Patients with the following diseases were excluded: (1) Blood system diseases; (2) Malignant tumors that have not been effectively treated; (3) Severe heart, liver, and spleen dysfunction; (4) Death within 2 months of onset. All cases were followed up using medical records and by telephone, and 141 patients with incomplete data and failure to follow‐up were removed from the study. The study was approved by the Ethics Committee of the First Affiliated Hospital of Guangxi Medical University (NO. 2022‐KY‐E‐(270)). All procedures performed in this study involving human participants were in accordance with the Declaration of Helsinki (as revised in 2013). Individual consent for this retrospective analysis was waived.

### 2.2 Laboratory data collection

At admission, blood samples were collected from patients for routine blood tests, erythrocyte sedimentation rate (ESR), and C‐reactive protein (CRP) examination. The examination reports were provided by the hospital laboratory, and the laboratory's normal values or reference ranges were used for comparison.

### 2.3 Statistical analysis

All statistical analyses were performed using SPSS version 25.0 (SPSS Inc.). Quantitative data were expressed as mean ± standard deviation and Student's *t* test was used to test the significance of differences between groups. Categorical variables were pooled using absolute frequencies and percentages, and the significance of differences between groups was calculated using one‐way analysis of variance or the *χ* ^2^ test. Univariate analysis (logistic regression) was used to identify variables associated with AP complications and to identify risk factors, followed by multivariate analysis using logistic regression and positive likelihood ratio test to identify independent risk factors closely associated with complications. Finally, the receiver operating curve (ROC) and area under the curve (AUC) were calculated to evaluate the ability of routine blood parameters to predict complications.

## 3 RESULTS

### 3.1 Clinical characteristics of study population

A total of 809 patients with complete data and complete follow‐up were included in the study, including 575 males and 234 females, with an average age of 50.94 ± 15.97 years. AP etiology included biliary tract disease (*n* = 336), alcoholism (*n* = 89), hyperlipidemia (*n* = 91), and others (*n* = 293). The main complications were: pancreatic pseudocyst, acute respiratory failure, sepsis, infectious pancreatic necrosis, acute renal failure. The details are listed in Table 1.

**Table 1:** Clinical characteristics of AP patients

Baseline characteristics	Acute pancreatitis (*n* = 809)
Sex (male/female)	575/234
Age (years)	50.94 ± 15.97
Etiology of AP (%)	
Biliary	336 (41.5%)
Alcoholic	89 (11.0%)
Hyperlipidemic	91 (11.2%)
Others	293 (35.3%)
Tobacco intake (sticks/day)	9.00 ± 12.62
First onset/Relapse	562/247
BMI (kg/m^2^)	23.63 ± 4.02
Diabetes (%)	133 (16.4%)
Stage (mild/moderate/severe) (%)	183 (22.6%)/424 (52.4%)/202 (25.0%)
Lab data	
White blood cell (10^9^/L)	11.84 ± 6.04
Neutrophil (10^9^/L)	9.45 ± 5.83
Lymphocyte (10^9^/L)	1.41 ± 0.78
Monocyte (10^9^/L)	0.75 ± 0.57
Eosinocyte (10^9^/L)	0.16 ± 0.31
Red blood cell (10^12^/L)	4.29 ± 0.97
Hemoglobin (g/L)	123.80 ± 27.16
Platelet (10^9^/L)	260.68 ± 119.36
Plateletocrit (%)	0.22 ± 0.10
Mean platelet volume (fL)	8.70 ± 1.42
C‐reactive protein (mg/L)	90.90 ± 83.23
Erythrocyte sedimentation rate (mm/60 min)	42.46 ± 29.39
Complications (%)	
Pancreatic pseudocyst	244 (30.2%)
Acute respiratory failure	70 (8.7%)
Sepsis	58 (7.2%)
Infectious pancreatic necrosis	55 (6.8%)
Acute renal failure	34 (4.2%)

*Note*: Data are expressed as mean ± SD and percentage.

Abbreviations: BMI, body mass index; SD, standard deviation.

### 3.2 Comparison of clinical characteristics of AP patients with complications and without complications

**Figure 1:**
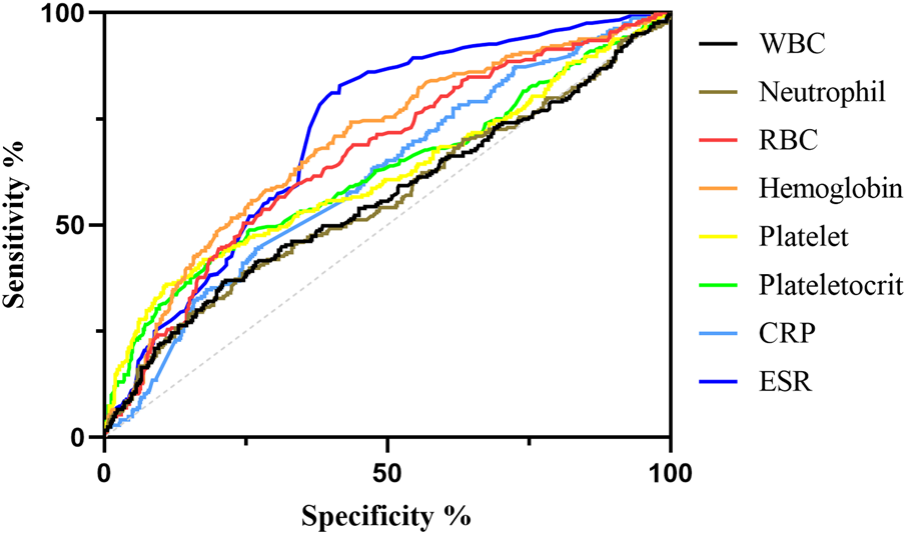
The receiver operating characteristic (ROC) curves of routine blood parameters for predicting pancreatic pseudocysts

**Figure 2:**
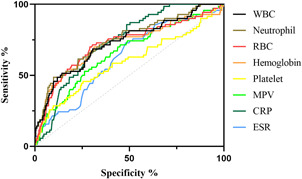
The receiver operating characteristic (ROC) curves of routine blood parameters for predicting acute respiratory failure

**Figure 3:**
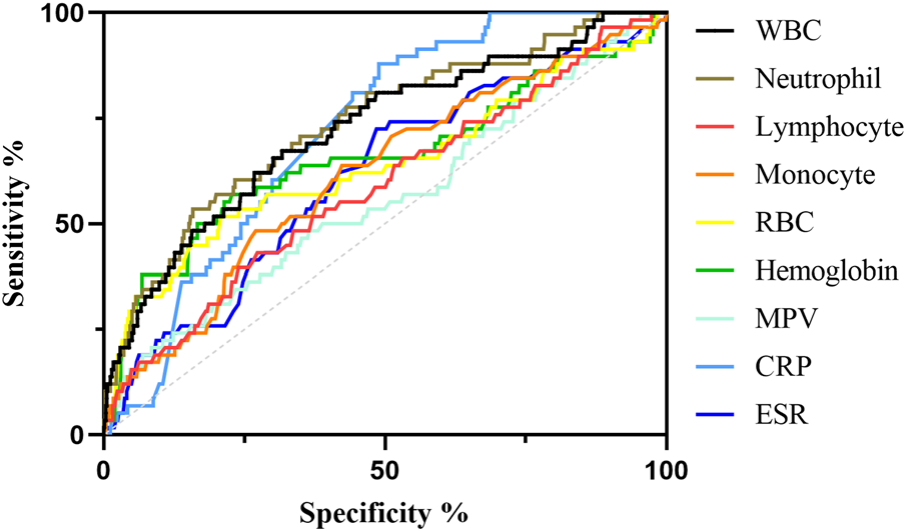
The receiver operating characteristic (ROC) curves of routine blood parameters for predicting sepsis

**Figure 4:**
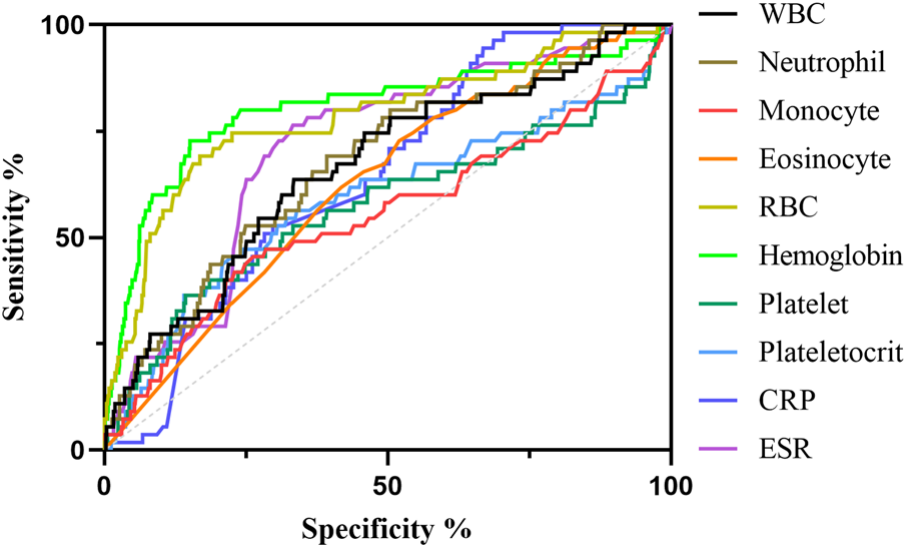
The receiver operating characteristic (ROC) curves of routine blood parameters for predicting infectious pancreatic necrosis

**Figure 5:**
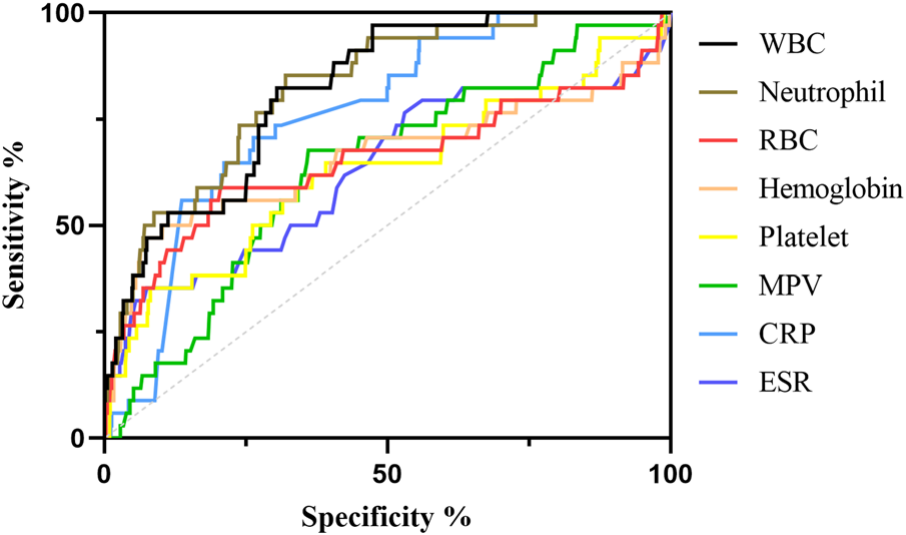
The receiver operating characteristic (ROC) curves of routine blood parameters for predicting acute renal failure

We included and analyzed five major complications. AP patients with pancreatic pseudocyst mostly had a history of heavy drinking and recurrence of pancreatitis, and most had lower body mass index (BMI) and severe pancreatitis. These patients had higher white blood cells (WBCs), neutrophils, CRP, ESR, PLT, and plateletocrit (PCT) on admission, and lower red blood cells (RBCs) and hemoglobin (Hb) (Table 2). ROC curve showed that among these routine blood parameters, ESR had the largest AUC (AUC = 0.718) and neutrophil had the smallest AUC (AUC = 0.558) (Table 3, Figure 1). AP patients with acute respiratory failure mostly had severe AP and first episode. These patients had higher WBCs, neutrophils, CRP, ESR, PLT, and mean platelet volume (MPV) on admission, and lower RBCs and Hb (Table 4). ROC curve showed that among these routine blood parameters, neutrophil had the largest AUC (AUC = 0.746) and PLT had the smallest AUC (AUC = 0.592) (Table 5, Figure 2). AP patients with sepsis mostly suffered from hyperlipidemia, and most of them had severe AP. These patients had higher WBCs, neutrophils, monocytes, MPV, CRP, and ESR on admission, and lower lymphocytes, RBCs, and Hb (Table 6). ROC curve showed that among these routine blood parameters, neutrophil had the largest AUC (AUC = 0.737) and MPV had the smallest AUC (AUC = 0.558) (Table 7, Figure 3). AP patients with infectious pancreatic necrosis mostly had severe pancreatitis with a history of heavy drinking. These patients had higher WBCs, neutrophils, monocytes, CRP, and ESR on admission, and lower eosinocyte, RBCs, and Hb (Table 8). ROC curve showed that among these routine blood parameters, Hb had the largest AUC (AUC = 0. 805) and monocyte had the smallest AUC (AUC = 0.554) (Table 9, Figure 4). AP patients with acute renal failure mostly had severe pancreatitis caused by other uncommon etiologies. These patients had higher WBCs, neutrophils, monocytes, MPV, CRP, and ESR on admission, and lower RBCs, Hb, and PLT (Table 10). ROC curve showed that among these routine blood parameters, neutrophil had the largest AUC (AUC = 0. 824) and PLT had the smallest AUC (AUC = 0. 627) (Table 11, Figure 5).

**Table 2:** Comparison of pseudocyst and non‐pseudocyst groups in AP patients

Baseline characteristics	Pseudocyst (*n* = 244)	Nonpseudocyst (*n* = 565)	*χ* ^2^/t	*p*
Sex (male/female)	184/60	391/174	3.193	.074
Age (years)	49.31 ± 14.13	51.64 ± 16.67	2.034	.042
Biliary (%)	86 (35.2%)	250 (44.2%)	5.687	.020
Alcoholic (%)	39 (16.0%)	50 (8.8%)	8.858	.005
Hyperlipidemic (%)	17 (7.0%)	74 (13.1%)	6.414	.011
Others (%)	102 (41.8%)	191 (33.8%)	4.719	.032
First onset (%)	152 (62.3%)	410 (72.6%)	8.476	.004
BMI (kg/m^2^)	22.90 ± 3.55	23.94 ± 4.18	3.406	.001
Stage (mild) (%)	9 (3.7%)	174 (30.8%)	71.541	<.001
Stage (moderate) (%)	126 (51.6%)	298 (52.7%)	0.083	.773
Stage (severe) (%)	109 (44.7%)	93 (16.5%)	72.396	<.001
White blood cell (10^9^/L)	13.05 ± 7.33	11.31 ± 5.30	3.795	<.001
Neutrophil (10^9^/L)	10.64 ± 7.14	8.94 ± 5.08	3.851	<.001
Lymphocyte (10^9^/L)	1.41 ± 0.85	1.41 ± 0.75	0.004	.997
Monocyte (10^9^/L)	0.79 ± 0.50	0.74 ± 0.60	1.113	.266
Eosinocyte (10^9^/L)	0.15 ± 0.25	0.16 ± 0.33	0.379	.705
Red blood cell (10^12^/L)	3.92 ± 0.93	4.44 ± 0.94	7.247	<.001
Hemoglobin (g/L)	111.68 ± 26.34	129.04 ± 25.83	8.717	<.001
Platelet (10^9^/L)	304.73 ± 150.35	241.65 ± 97.31	7.107	<.001
Plateletocrit (%)	0.26 ± 0.12	0.21 ± 0.08	7.019	<.001
Mean platelet volume (fL)	8.57 ± 1.30	8.76 ± 1.47	1.802	.072
C‐reactive protein (mg/L)	111.54 ± 86.16	81.99 ± 80.38	4.695	<.001
Erythrocyte sedimentation rate (mm/60 min)	57.18 ± 28.34	36.10 ± 27.52	9.913	<.001

**Table 3:** Efficacy of routine blood parameters in predicting pseudocysts

Name	Sensitivity (%)	Specificity (%)	Odds ratio	AUC (95% CI)	Threshold
White blood cell (10^9^/L)	36.63	79.12	1.754	0.560 (0.514−0.605)	>14.76
Neutrophil (10^9^/L)	38.93	75.22	1.571	0.558 (0.513−0.603)	>11.63
Red blood cell (10^12^/L)	50.41	75.4	2.049	0.661 (0.620−0.702)	<3.965
Hemoglobin (g/L)	74.18	56.46	1.704	0.691 (0.652−0.731)	<127.6
Platelet (10^9^/L)	36.07	89.03	3.287	0.615 (0.569−0.660)	>338.5
Plateletocrit (%)	48.77	74.34	1.900	0.620 (0.575−0.664)	>0.2435
C‐reactive protein (mg/L)	44.26	73.27	1.656	0.612 (0.570−0.653)	>104.9
Erythrocyte sedimentation rate (mm/60 min)	82.79	58.41	1.99	0.718 (0.681−0.754)	>34.50

*Note*: AUC is the area under the ROC curve, and the range of 95% CI is shown. Odds ratio was calculated as %sensitivity × %specificity/(100−%sensitivity) × (100−%specificity).

Abbreviations: AUC, area under the curve; CI, confidence interval; ROC, receiver operating curve.

**Table 4:** Comparison of AP patients with acute respiratory failure and nonacute respiratory failure groups

Baseline characteristics	Acute respiratory failure (*n* = 70)	Nonacute respiratory failure (*n* = 739)	*χ* ^2^/t	*p*
Sex (male/female)	49/21	526/213	0.043	.836
Age (years)	50.61 ± 17.32	50.97 ± 15.85	0.177	.860
Biliary (%)	23 (32.9%)	313 (42.4%)	2.375	.123
Alcoholic (%)	8 (11.4%)	81 (11.0%)	0.014	.905
Hyperlipidemic (%)	10 (14.3%)	81 (11.0%)	0.708	.400
Others (%)	29 (41.4%)	264 (35.7%)	0.901	.343
First onset (%)	58 (82.9%)	504 (68.2%)	6.476	.010
BMI (kg/m^2^)	24.42 ± 4.67	23.55 ± 3.95	1.714	.097
Stage (mild) (%)	0 (0.0%)	183 (24.8%)	22.402	<.001
Stage (moderate) (%)	14 (20%)	410 (55.5%)	32.273	<.001
Stage (severe) (%)	56 (80%)	146 (19.8%)	123.872	<.001
White blood cell (10^9^/L)	17.65 ± 9.22	11.29 ± 5.33	8.820	<.001
Neutrophil (10^9^/L)	15.37 ± 8.76	8.89 ± 5.13	9.358	<.001
Lymphocyte (10^9^/L)	1.28 ± 0.96	1.43 ± 0.76	1.491	.136
Monocyte (10^9^/L)	0.87 ± 0.67	0.74 ± 0.56	1.758	.079
Eosinocyte (10^9^/L)	0.10 ± 0.22	0.16 ± 0.31	1.593	.111
Red blood cell (10^12^/L)	3.58 ± 1.09	4.35 ± 0.93	6.538	<.001
Hemoglobin (g/L)	104.58 ± 33.90	125.62 ± 25.73	6.344	<.001
Platelet (10^9^/L)	233.78 ± 130.52	263.41 ± 117.98	2.124	.034
Plateletocrit (%)	0.21 ± 0.12	0.22 ± 0.09	0.639	.523
Mean platelet volume (fL)	9.44 ± 1.57	8.63 ± 1.39	4.617	<.001
C‐reactive protein (mg/L)	147.07 ± 76.07	85.58 ± 81.95	6.036	<.001
Erythrocyte sedimentation rate (mm/60 min)	55.11 ± 32.48	41.26 ± 28.82	3.801	<.001

**Table 5:** Efficacy of routine blood parameters in predicting acute respiratory failure

Name	Sensitivity (%)	Specificity (%)	Odds ratio	AUC (95% CI)	Threshold
White blood cell (10^9^/L)	68.57	69.42	2.242	0.729 (0.662−0.795)	>13.10
Neutrophil (10^9^/L)	48.57	90.12	4.917	0.746 (0.682−0.810)	>15.56
Red blood cell (10^12^/L)	71.43	69.55	2.346	0.720 (0.649−0.791)	<4.01
Hemoglobin (g/L)	70	68.74	2.239	0.714 (0.642−0.787)	<116.2
Platelet (10^9^/L)	45.71	74.56	1.797	0.592 (0.513−0.671)	<190.1
Mean platelet volume (fL)	52.86	73.75	2.013	0.658 (0.589−0.727)	>9.415
C‐reactive protein (mg/L)	85.71	51.42	1.764	0.736 (0.685−0.786)	>74.48
Erythrocyte sedimentation rate (mm/60 min)	72.86	51.96	1.517	0.626 (0.560−0.693)	>38.50

Abbreviations: AUC, area under the curve; CI, confidence interval.

**Table 6:** Comparison of sepsis and nonsepsis groups in AP patients

Baseline characteristics	Sepsis (*n* = 58)	Nonsepsis (*n* = 751)	*χ* ^2^/t	*p*
Sex (male/female)	44/14	531/220	0.696	.404
Age (years)	50.98 ± 19.31	50.93 ± 15.70	0.023	.982
Biliary (%)	20 (34.5%)	316 (42.1%)	1.279	.258
Alcoholic (%)	3 (5.2%)	86 (11.5%)	2.168	.141
Hyperlipidemic (%)	12 (20.7%)	79 (10.5%)	5.579	.028
Others (%)	23 (39.7%)	270 (36.0%)	0.320	.572
First onset (%)	43 (74.1%)	519 (69.1%)	0.642	.423
BMI (kg/m^2^)	24.46 ± 5.07	23.57 ± 3.93	1.627	.104
Stage (mild) (%)	4 (6.9%)	179 (23.8%)	8.825	.002
Stage (moderate) (%)	20 (34.5%)	404 (53.8%)	8.051	.006
Stage (severe) (%)	34 (58.6%)	168 (22.4%)	37.766	<.001
White blood cell (10^9^/L)	17.56 ± 9.22	11.41 ± 5.49	7.756	<.001
Neutrophil (10^9^/L)	15.24 ± 8.90	9.00 ± 5.27	8.166	<.001
Lymphocyte (10^9^/L)	1.21 ± 0.61	1.43 ± 0.79	2.101	.036
Monocyte (10^9^/L)	0.98 ± 0.77	0.73 ± 0.55	3.239	.001
Eosinocyte (10^9^/L)	0.10 ± 0.22	0.16 ± 0.31	1.439	.151
Red blood cell (10^12^/L)	3.77 ± 1.22	4.33 ± 0.93	4.263	<.001
Hemoglobin (g/L)	107.92 ± 33.81	124.86 ± 26.60	4.577	<.001
Platelet (10^9^/L)	233.11 ± 124.14	262.81 ± 118.80	1.828	.068
Plateletocrit (%)	0.20 ± 0.09	0.22 ± 0.10	1.753	.080
Mean platelet volume (fL)	9.08 ± 1.67	8.67 ± 1.40	2.124	.034
C‐reactive protein (mg/L)	140.15 ± 68.65	87.10 ± 83.08	4.739	<.001
Erythrocyte sedimentation rate (mm/60 min)	53.31 ± 31.15	41.62 ± 29.11	2.932	.003

**Table 7:** Efficacy of routine blood parameters in predicting sepsis

Name	Sensitivity (%)	Specificity (%)	Odds ratio	AUC (95% CI)	Threshold
White blood cell (10^9^/L)	67.24	68.31	2.122	0.722 (0.650−0.794)	>13.07
Neutrophil (10^9^/L)	53.45	84.15	3.373	0.737 (0.667−0.806)	>13.72
Lymphocyte (10^9^/L)	43.1	72.7	1.579	0.585 (0.506‐0.661)	<0.975
Monocyte (10^9^/L)	63.79	57.66	1.507	0.617 (0.541−0.693)	>0.695
Red blood cell (10^12^/L)	51.72	79.09	2.474	0.639 (0.551−0.727)	<3.63
Hemoglobin (g/L)	56.9	77.23	2.499	0.657 (0.570−0.744)	<106.3
Mean platelet volume (fL)	20.69	91.48	2.428	0.558 (0.476−0.640)	>10.65
C‐reactive protein (mg/L)	87.93	51.13	1.799	0.718 (0.666−0.771)	>75.29
Erythrocyte sedimentation rate (mm/60 min)	72.41	51.53	1.494	0.613 (0.538−0.689)	>38.50

Abbreviations: AUC, area under the curve; CI, confidence interval.

**Table 8:** Comparison of infectious pancreatic necrosis and noninfectious pancreatic necrosis groups in AP patients

Baseline characteristics	Infectious pancreatic necrosis (*n* = 55)	Noninfectious pancreatic necrosis (*n* = 754)	χ^2^/t	*p*
Sex (male/female)	41/14	534/220	0.346	.557
Age (years)	47.38 ± 17.46	51.20 ± 15.84	1.712	.087
Biliary (%)	16 (29.1%)	320 (42.4%)	3.762	.052
Alcoholic (%)	11 (20.0%)	78 (10.3%)	4.881	.041
Hyperlipidemic (%)	4 (7.3%)	87 (11.5%)	0.934	.334
Others (%)	24 (43.6%)	269 (35.7%)	1.406	.236
First onset (%)	43 (78.2%)	519 (68.8%)	2.112	.146
BMI (kg/m^2^)	23.94 ± 4.05	23.61 ± 4.02	0.593	.553
Stage (mild) (%)	0 (0.0%)	183	17.251	<.001
Stage (moderate) (%)	11 (20%)	413 (54.8%)	24.853	<.001
Stage (severe) (%)	44 (80%)	158 (21.0%)	95.392	<.001
White blood cell (10^9^/L)	15.68 ± 8.17	11.56 ± 5.76	4.962	<.001
Neutrophil (10^9^/L)	13.25 ± 7.81	9.17 ± 5.56	5.088	<.001
Lymphocyte (10^9^/L)	1.28 ± 0.72	1.42 ± 0.79	1.297	.195
Monocyte (10^9^/L)	0.92 ± 0.88	0.74 ± 0.54	2.279	.023
Eosinocyte (10^9^/L)	0.07 ± 0.10	0.16 ± 0.32	2.104	.036
Red blood cell (10^12^/L)	3.28 ± 1.02	4.36 ± 0.92	8.360	<.001
Hemoglobin (g/L)	93.28 ± 31.01	126.03 ± 25.49	9.055	<.001
Platelet (10^9^/L)	298.70 ± 165.51	257.90 ± 114.94	2.455	.014
Plateletocrit (%)	0.25 ± 0.11	0.22 ± 0.09	2.258	.024
Mean platelet volume (fL)	8.73 ± 1.48	8.70 ± 1.42	0.130	.896
C‐reactive protein (mg/L)	119.06 ± 69.61	88.84 ± 83.81	2.609	.009
Erythrocyte sedimentation rate (mm/60 min)	63.85 ± 30.11	40.90 ± 28.74	5.700	<.001

**Table 9:** Efficacy of routine blood parameters in predicting infectious pancreatic necrosis

Name	Sensitivity (%)	Specificity (%)	Odds ratio	AUC (95% CI)	Threshold
White blood cell (10^9^/L)	63.64	66.58	1.904	0.666 (0.590−0.741)	>12.75
Neutrophil (10^9^/L)	69.09	60.74	1.760	0.675 (0.601−0.749)	>9.47
Monocyte (10^9^/L)	45.45	73.74	1.731	0.554 (0.464−0.644)	>0.875
Eosinocyte (10^9^/L)	72.73	48.01	1.399	0.624 (0.553−0.695)	<0.085
Red blood cell (10^12^/L)	69.09	83.16	4.102	0.784 (0.712−0.855)	<3.56
Hemoglobin (g/L)	72.73	84.88	4.810	0.805 (0.730−0.879)	<99.25
Platelet (10^9^/L)	36.36	85.68	2.539	0.572 (0.479−0.666)	>361.6
Plateletocrit (%)	45.45	77.45	2.016	0.592 (0.502−0.683)	>0.270
C‐reactive protein (mg/L)	98.18	29.58	1.394	0.646 (0.584−0.708)	>18.82
Erythrocyte sedimentation rate (mm/60 min)	76.36	66.71	2.294	0.713 (0.646−0.779)	>49.50

Abbreviations: AUC, area under the curve; CI, confidence interval.

**Table 10:** Comparison of AP patients with acute renal failure and nonacute renal failure groups

Baseline characteristics	Acute renal failure (*n* = 34)	Nonacute renal failure (*n* = 775)	*χ* ^2^/t	*p*
Sex (male/female)	26/8	549/226	0.503	.478
Age (years)	47.47 ± 16.07	51.09 ± 15.96	1.293	.196
Biliary (%)	4 (11.8%)	332 (42.8%)	12.952	<.001
Alcoholic (%)	5 (14.7%)	84 (10.8%)	0.498	.481
Hyperlipidemic (%)	3 (8.8%)	88 (11.4%)	0.209	.648
Others (%)	22 (64.7%)	271 (35.0%)	12.469	.001
First onset (%)	25 (73.5%)	537 (69.3%)	0.276	.599
BMI (kg/m^2^)	24.11 ± 3.25	23.61 ± 4.05	0.707	.480
Stage (mild) (%)	0 (0.0%)	183 (73.5%)	10.375	.001
Stage (moderate) (%)	7 (20.6%)	417 (53.8%)	14.410	<.001
Stage (severe) (%)	27 (79.4%)	175 (22.6%)	56.152	<.001
White blood cell (10^9^/L)	20.63 ± 10.06	11.49 ± 5.56	8.971	<.001
Neutrophil (10^9^/L)	18.03 ± 9.64	9.07 ± 5.30	9.219	<.001
Lymphocyte (10^9^/L)	1.44 ± 1.02	1.41 ± 0.77	0.169	.865
Monocyte (10^9^/L)	0.98 ± 0.84	0.74 ± 0.55	2.359	.019
Eosinocyte (10^9^/L)	0.15 ± 0.30	0.16 ± 0.31	0.143	.886
Red blood cell (10^12^/L)	3.72 ± 1.40	4.31 ± 0.94	3.543	<.001
Hemoglobin (g/L)	107.33 ± 41.90	124.52 ± 26.13	3.640	<.001
Platelet (10^9^/L)	221.08 ± 141.43	262.41 ± 118.10	1.980	.048
Plateletocrit (%)	0.20 ± 0.14	0.22 ± 0.09	1.151	.250
Mean platelet volume (fL)	9.26 ± 1.36	8.68 ± 1.42	2.321	.021
C‐reactive protein (mg/L)	158.24 ± 72.49	87.94 ± 82.46	4.888	<.001
Erythrocyte sedimentation rate (mm/60 min)	60.15 ± 40.76	41.68 ± 28.58	3.612	<.001

**Table 11:** Efficacy of routine blood parameters in predicting acute renal failure

Name	Sensitivity (%)	Specificity (%)	Odds ratio	AUC (95% CI)	Threshold
White blood cell (10^9^/L)	82.35	69.55	2.704	0.817 (0.756−0.878)	>13.32
Neutrophil (10^9^/L)	85.29	68	2.665	0.824 (0.760−0.889)	>10.46
Red blood cell (10^12^/L)	58.82	79.48	2.867	0.651 (0.529−0.774)	<3.575
Hemoglobin (g/L)	47.06	92.9	6.631	0.658 (0.534−0.782)	<83.55
Platelet (10^9^/L)	35.29	91.87	4.342	0.627 (0.515−0.739)	<134.6
Mean platelet volume (fL)	67.65	64	1.879	0.634 (0.542−0.727)	>8.985
C‐reactive protein (mg/L)	70.59	73.68	2.682	0.762 (0.693−0.830)	>120.5
Erythrocyte sedimentation rate (mm/60 min)	35.29	92.52	4.716	0.629 (0.515−0.742)	>86.50

Abbreviations: AUC, area under the curve; CI, confidence interval.

### 3.3 Univariate and multivariate predictive factor analyses of complications in AP patients

Because the incidence of acute respiratory failure, sepsis, infectious pancreatic necrosis, acute renal failure complications were less than 10%, we focused on the analysis of AP patients with pancreatic pseudocyst complications. To further evaluate the predictive power of routine blood parameters for pseudocysts, we performed univariate and multivariate logistic regression analyses. Parameters, such as WBC, neutrophil, RBC, Hb, PLT, PCT, CRP, ESR were introduced, and potential confounding factors were considered, including gender, age, etiology, number of episodes, BMI, and severity of disease. As shown in Table 12, after adjusting the prognostic factors by multivariate logistic regression, first onset, BMI, Hb, PLT, and ESR were independent predictors of pseudocyst, of which PLT and ESR were independent risk factors. AP patients with recurrence, lower BMI and Hb, and higher PLT and ESR were more likely to develop pseudocysts.

**Table 12:** Univariate and multivariate logistic regression analysis of the predictive factors for pseudocysts

			Univariate analysis			Multivariate analysis		
Variable	Classification	Number of patients	OR	95% CI	*p*	OR	95% CI	*p*
Sex	Male/female	575/234	1.365	0.970−1.921	.074	0.799	0.513−1.243	.320
Age (yrs)	<69/≥69	685/124	0.537	0.337−0.857	.008	0.711	0.393−1.285	.259
Etiology	Alcoholic/other	89/720	1.960	1.251−3.070	.003	1.783	0.995−3.194	.052
Onset	First onset/relapse	562/247	0.625	0.454−0.859	.004	0.597	0.400−0.890	.011
BMI (kg/m^2^)	<25.74/≥25.74	599/210	0.456	0.310−0.669	<.001	0.159	0.097−0.259	<.001
Stage	Severe/other	202/607	4.098	2.927−5.736	<.001	0.969	0.605−1.552	.895
WBC (10^9^/L)	<14.77 × 10^9^/≥14.77 × 10^9^	602/207	2.175	1.563−3.026	<.001	1.925	0.864−4.29	.109
NE (10^9^/L)	<11.64 × 10^9^/≥11.64 × 10^9^	574/235	1.936	1.405−2.667	<.001	0.904	0.414−1.977	.801
RBC (10^12^/L)	<3.97 × 10^12^/≥3.97 × 10^12^	262/547	0.321	0.234−0.440	<.001	0.791	0.492−1.272	.333
Hb (g/L)	<127.6/≥127.6	427/382	0.268	0.193−0.374	<.001	0.426	0.264−0.689	.001
PLT (10^9^/L)	<338.6 × 10^9^/≥338.6 × 10^9^	659/150	4.577	3.157−6.634	<.001	2.96	1.64−5.342	<.001
PCT (%)	<0.245/≥0.245	548/261	2.764	2.018−3.786	<.001	1.238	0.746−2.053	.409
CRP (mg/L)	<105.0/≥105.0	550/259	2.177	1.591−2.980	<.001	1.417	0.94−2.134	.096
ESR (mm/60 min)	<35/≥35	372/437	6.754	4.655−9.798	<.001	6.883	4.184−11.325	<.001

Abbreviations: CRP, C‐reactive protein; ESR, Erythrocyte sedimentation rate; Hb, Hemoglobin;  NE, Neutrophilicgranulocyte; PCT, Plateletocrit; PLT, Platelet; RBC, Red blood cell; WBC, White blood cell.

### 3.4 Establishment and validation of routine blood parameter prediction system

**Figure 6:**
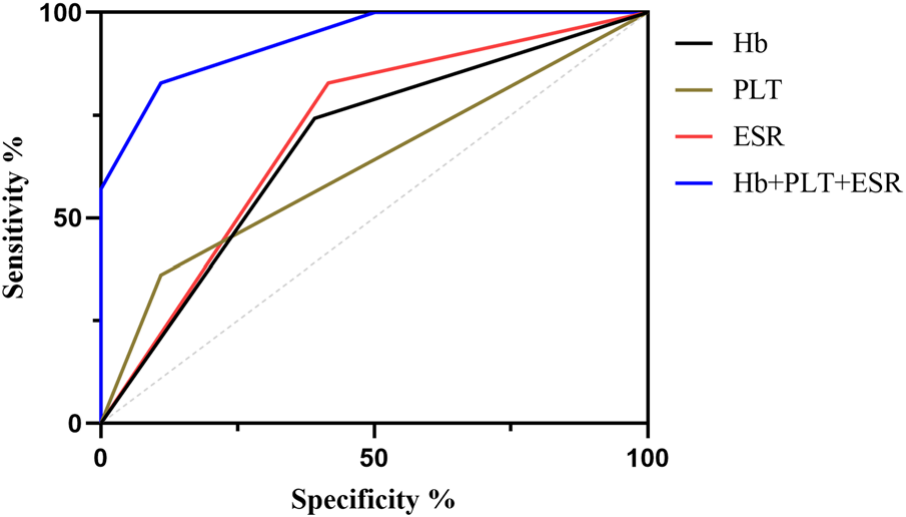
The receiver operating characteristic (ROC) curves of Hb, PLT, and ESR for predicting pancreatic pseudocyst alone and in combination. ESR, Erythrocyte sedimentation rate; Hb, Hemoglobin; PLT, Platelet.

From the above data, we found that blood tests routinely used for AP patients in the clinical setting could act as predictors of pancreatic pseudocysts. To optimize the predictive ability, we constructed a predictive model jointly composed of ESR, PLT, and Hb. As shown in Figure 6, AP patients with higher ESR, PLT, and lower Hb are more likely to develop pseudocyst. Our data suggest that the combination of these three parameters can improve the accuracy of prediction and may be an effective tool for identifying the risk of pseudocyst development in AP patients (Table 13).

**Table 13:** Efficacy of Hb, PLT and ESR in predicting pancreatic pseudocysts

Name	Sensitivity (%)	Specificity (%)	Odds ratio	AUC (95%CI)	Threshold
Hb (g/L)	74.18	56.46	1.704	0.691 (0.652−0.731)	<127.6
PLT (10^9^/L)	36.07	89.03	3.287	0.615 (0.569−0.660)	>338.5
ESR (mm/60 min)	82.79	58.41	1.99	0.718 (0.681−0.754)	>34.50
Hb+PLT + ESR	82.79	89.03	7.544	0.933 (0.916−0.951)	‐

Abbreviations: ESR, Erythrocyte sedimentation rate; Hb, Hemoglobin; PLT, Platelet.

## 4 DISCUSSION

AP is an inflammatory process of the pancreatic gland, accompanied by the release of large amounts of amylase and lipase into the blood circulation, often leading to local and systemic complications, and can lead to multiple organ failure and death. Most AP patients have a mild course of disease, but nearly 20% of patients can experience complications, and those with complications have a mortality rate as high as 60%.^7^, ^16^, ^17^ Most complications cannot be treated by specific drugs; some require surgical intervention, such as endoscopic surgery, and are prone to recurrence after surgery, which adversely affects the quality of life of patients, increasing the economic and psychological burden. Therefore, early prediction of the possible complications in AP patients and early intervention can improve their condition and prognosis. Although some AP complication prediction models are considered effective,^18^, ^19^, ^20^ these models involve some complex and expensive examinations that many medical institutions cannot implement, resulting in limited clinical application.

Routine blood test is a very simple and inexpensive test that can be performed in almost all medical institutions, and is usually used to determine the degree of inflammation and infection.^13^, ^21^, ^22^, ^23^, ^24^ AP is a disease that causes local or systemic inflammatory response; hence, it is desirable to use routine blood tests to predict the severity of AP and its complications. Our study found that AP patients with complications, such as pseudocyst, acute respiratory failure, sepsis, infectious pancreatic necrosis, and acute renal failure had significantly increased inflammatory indicators, such as WBCs, neutrophils, CRP, and ESR, while RBCs, Hb decreased significantly, indicating that the pathological basis of AP complications is an inflammatory response, and malnutrition and ischemia and hypoxia may further aggravate this inflammatory response.

Although routine blood tests are effective in predicting complications of AP, specificity needs to be improved, as these indicators have also been reported to be useful in predicting diseases other than those of the pancreas.^13^, ^25^, ^26^, ^27^ The ESR measures the rate at which RBCs settle in the plasma of anticoagulated blood in a standardized Westergren tube, and is often used as a measure of infection, rheumatic autoimmune disease; however, noninflammatory factors can also affect ESR.^28^, ^29^ Electric charge is the main cause of ESR changes, and positively charged proteins, such as immunoglobulin and fibrinogen in plasma can lead to increased ESR.^30^ CRP is an acute phase response protein synthesized by the liver and an important indicator that measures the severity of the inflammatory response.^31^ PLT mainly plays a decisive role in thrombosis and hemostasis, but also plays an important role in other disorders, including inflammation, disorders of immunity, tumors.^32^, ^33^ Interestingly, after introducing other confounding factors, we found that none of the factors representing acute inflammatory responses like WBCs, neutrophils, and CRP were independent predictors of pseudocysts, whereas ESR and PLTs were independent predictors, indicating that pancreatic pseudocyst is not caused by a simple acute inflammatory reaction, but is closely related to multiple complex factors, including chronic inflammation of pancreas. Multivariate analysis also showed that patients with relapse were more likely to develop pseudocysts than patients with first‐episode AP; this is consistent with previous studies.^34^, ^35^, ^36^ To improve the accuracy of predicting AP complications, we combined some routine blood parameters. We found that AP patients with higher ESR and PLT and lower Hb had a higher risk of pseudocysts, and the combination of the three parameters had better predictive ability for pseudocysts. Unfortunately, due to the low incidence of acute respiratory failure, sepsis, infectious pancreatic necrosis, and acute renal failure complications, we were unable to perform a multivariate analysis and therefore could not build a joint predictive model.

As a systemic inflammatory disease, AP can present with multiple complications.^9^, ^37^, ^38^ Most complications occur within 2 weeks of onset, whereas pancreatic pseudocysts usually occur around 4 weeks after onset. At this time, most patients have been discharged, and the early stage of pseudocyst formation often lacks characteristic symptoms, which can easily lead to delayed diagnosis and affect treatment. Therefore, early prediction of the occurrence of pseudocysts is particularly important. Previous studies have shown that male sex, smoking, and alcohol etiology are risk factors for pancreatic pseudocyst development. Alcohol causes chronic pancreatitis and pseudocysts through direct toxic effects on pancreatic acinar and ductal cells.^36^, ^39^, ^40^ However, our study did not show that the occurrence of pseudocysts was related to gender and smoking. Univariate analysis showed an association with alcohol etiology (*p* = .005), but multivariate analysis incorporating other confounding factors did not show a significant association (*p* = .052). Similarly, Szentesi et al.^41^ and Anahita et al.^42^ suggested that diabetes and obesity may be risk factors for pseudocyst development, but our study did not support this view, and the differences in these results may be related to regional population differences or medical history and collection deviations.

Although 809 AP patients were included in our study, this number is still a low proportion relative to the large number of AP patients. Moreover, our study was retrospective, and there was uncontrollability in medical history collection and population follow‐up, so there may be some limitations and biases. Our findings need to be corroborated and validated in prospective multicenter studies with larger cohort size.

## 5 CONCLUSIONS

We assessed the ability of routine blood parameters to predict complications in AP patients. We found that WBCs, neutrophils, RBCs, Hb, PLTs, CRP, and ESR appeared to be promising predictors of common complications in AP. These biomarkers are simple and easy to assess, have low cost and good reproducibility, which cannot only reduce the economic burden of patients, but also help clinicians, especially physicians in primary medical institutions, better manage AP patients.

## CONFLICT OF INTEREST

The authors declare no conflict of interest.
